# Microgeometrical dendritic factors predict electrical decoupling between somatic and dendritic compartments in magnocellular neurosecretory neurons

**DOI:** 10.3389/fncel.2023.1125029

**Published:** 2023-03-24

**Authors:** Sergiy M. Korogod, Javier E. Stern, Gennady S. Cymbalyuk

**Affiliations:** ^1^The Neuroscience Institute, Georgia State University, Atlanta, GA, United States; ^2^Department of Molecular Biophysics, O. O. Bogomoletz Institute of Physiology, National Academy of Sciences of Ukraine, Kyiv, Ukraine; ^3^Center for Neuroinflammation and Cardiometabolic Diseases, Georgia State University, Atlanta, GA, United States

**Keywords:** dendritic release, varicosity, glial sheath wrapping, peri-dendritic space, dendritic voltage attenuation, electro-geometrical (de)coupling

## Abstract

It is generally assumed that dendritic release of neuropeptides from magnocellular neurosecretory neurons (MNNs), a critical process involved in homeostatic functions, is an activity-dependent process that requires backpropagating action potentials (APs). Still, growing evidence indicates that dendritic release can occur in the absence of APs, and axonal APs have been shown to fail to evoke dendritic release. These inconsistencies strongly suggest that APs in MNNs may fail to backpropagating into dendrites. Here we tested whether simple factors of electrical signal attenuation could lead to effective decoupling between cell’s body and dendritic release site within typical geometrical characteristics of MNN. We developed a family of linear mathematical models of MNNs and evaluated whether the somato-dendritic transfer of electrical signals is influenced by the geometrical characteristics. We determined the prerequisites for critically strong dendritic attenuation of the somatic input which are sufficient to explain the failure of APs initiated in the soma to backpropagating into dendritic compartments. Being measured in 100 μm from soma voltage attenuations down to 0.1 and 0.01 of the input value were chosen as the markers of electrical decoupling of dendritic sites from the soma, considering 0.1 insufficient for triggering dendritic spikes and 0.01 indistinguishable from background noise. The tested micro-geometrical factors were the dendritic stem diameter, varicosities, and size of peri-dendritic space limited by glial sheath wrapping. Varicosities increased the attenuation along homogeneous proximal dendrites by providing an increased current leak at the junction with the proximal dendritic section. The glial sheath wrapping a dendrite section promoted greater attenuation by increasing longitudinal resistance of the interstitial peri-dendritic space thus playing the insulating role. These decoupling effects were strengthened in the case of the dendritic stems with thinner diameters of and/or increased conductivity of the membrane. These micro-geometrical factors are biophysically realistic and predict electrical decoupling between somatic and dendritic compartments in MNNs.

## 1. Introduction

Magnocellular neurosecretory neurons (MNNs) in the supraoptic (SON) and paraventricular (PVN) hypothalamic nuclei synthesize and secrete the neuropeptides oxytocin (OT) and vasopressin (VP). In addition to being released from axonal terminals in the posterior pituitary, both neuropeptides are also released from somato-dendritic compartments (Brown et al., 2013, 2020; Ludwig and Stern, 2015). Dendritic release subserves distinct functions from those involved in systemic release, and these include autoregulation of MNNs activity, modulation of local synaptic efficacy, and interpopulation communication (Gouzènes et al., 1998; Hermes et al., 2000; Hirasawa et al., 2004; Son et al., 2013). A large body of evidence supports dendritic release to occur in an activity-dependent manner and in response to physiologically relevant stimuli, such as the release of VP triggered by an increase in plasma osmolality (Sato et al., 2011), and the release of OT induced by suckling or by the increase of melanocyte stimulating hormone (aMSH) (Bealer and Crowley, 1998; Sabatier et al., 2003; Sabatier and Leng, 2006). Still, there is conflicting information in the literature regarding whether dendritic release depends on action potentials invading retrogradely dendritic compartments (e.g., action potential backpropagating), as shown for other neurons in the brain (Bergquist and Ludwig, 2008). For example, neuropeptide dendritic release can occur in the absence of action potentials (Son et al., 2013; Pitra et al., 2019) and independently of axonal release (Ludwig et al., 2002; Sabatier et al., 2003). Moreover, a study by Ludwig et al. showed that antidromic stimulation of axons in the pituitary failed to evoke dendritic release of VP unless the SON was primed with thapsigargin (Ludwig et al., 2005). This is functionally important because it further supports the notion that in MNNs, release from dendritic and axonal compartments can be evoked and regulated independently (Ludwig and Leng, 2006). The most striking example of this is represented by aMSH actions on oxytocin MNNs, which acting on MC4 receptors, it evokes dendritic release of oxytocin, while simultaneously inhibiting their firing activity, thus reducing systemic axonal release of the same neuropeptide (Sabatier et al., 2003). In addition, we recently showed that during pregnancy and lactation, aMSH-evoked axonal (but not dendritic) release of oxytocin was affected (Perkinson et al., 2022), further supporting a differential regulation dendritic vs. axonal release of the same peptide in the same neuron. Finally, action potential-independent dendritic release suggests that dendritic release in MNNs may occur in response to signals generated locally within the dendrites themselves.

One possible explanation for these inconsistent results is that under a basal physiological state, spikes fail to invade dendritic compartments, indicative of an electrical decoupling of SON dendrites from the spike initiation zone in the soma/axonal hillock.

Compared to other hypothalamic neurons including preautonomic (Stern, 2001), perinuclear zone (Armstrong and Stern, 1997) and GnRH neurons (Campbell et al., 2005; Herbison, 2021), MNNs have unique and distinctive morphological features, including a relatively large soma [dimensions up to 25–30 μm by 16–17 μm, membrane area ∼1,100–1,500 μm^2^ (Stern and Armstrong, 1998; Chen et al., 2022)] from where a relatively simple dendritic arborization emerges. MNNs have typically 1–3 primary dendrites (Stern and Armstrong, 1998; Chen et al., 2022) that ramified on average to no more than six levels (Stern and Armstrong, 1998) and have path lengths up to 300–500 μm (Stern and Armstrong, 1998). Moreover, MNNs contain specialized enlargements or swellings named varicosities (Stern, 2001; Ludwig and Stern, 2015), in which most of the neuropeptide cargo is stored, and from where release is believed to occur (Ludwig and Leng, 2006). These varicosities can often reach diameters up to 15 μm as shown both in fixed tissue (Ludwig and Leng, 2006) and more recently *in vivo* (Roy et al., 2021). Still, whether dendritic varicosities have an impact on electrotonic properties, and to what extent they could affect the ability of electrical signals to propagate between neuronal compartments is largely unknown.

Using computer modeling and the theory of electric circuits, we aimed in this study to explore possible and unique morphological aspect of SON MNNs, in particular the presence of dendritic varicosities, to assess their impact on the ability of electrical signals to propagate from the soma to dendritic compartments of MNNs.

In neurons, including MNNs, structural elements (axon, soma, dendrites) are electrically coupled by lateral currents, which flow in the intracellular and extracellular spaces (ICS and ECS, respectively) through conductive fluids having purely passive, linear ohmic properties (Jack et al., 1975; Segev and London, 2000). The ICS and ECS are shaped by the microgeometry of the neuron under study and surrounding neuronal and non-neuronal cells, including different type glial cells (Syková, 2004; Theodosis et al., 2008). Among the latter, the astrocytes, the most abundant type in the adult brain (Kettenmann and Ransom, 2005; Jäkel and Dimou, 2017), are of particular interest being as they are known for “wrapping” hypothalamic neurons by their processes, and this micromorphological feature changes depending on physiological and pathological conditions (Hatton, 1999; Langle et al., 2002; Theodosis et al., 2008). Consequences of such remodeling of neuron-glia microgeometry for neurons functioning remain poorly understood and thus attracted our attention in this study referring exclusively to astrocytes (and using general term “glia” as a synonym). Based on available data describing MNN microgeometry (Smith and Armstrong, 1990; Stern and Armstrong, 1998; Chen et al., 2022), we built biologically inspired cell models and explored geometry-related features of the somato-dendritic passive transfer of voltages and currents. To derive and solve model equations, we used methods of the theory of electro-geometrical coupling and parametric sensitivity of the dendritic transfer functions, which were systematically described earlier (Korogod and Kaspirzhny, 2008, 2011; Korogod and Tyc-Dumont, 2009). Using this approach, we specify how key cellular parameters, individually and collectively, influence the ability of electrical signals to backpropagate from the soma to dendritic compartments. These include dendritic stem diameter, varicosities, and size of the peri-dendritic space limited by astrocytic sheaths.

Under physiological conditions, MNN are tightly enwrapped by astrocyte processes to make the under-wrap peri-dendritic space very small. The obvious biophysical consequences of that are an increased resistance of a thinner extracellular fluid volume conductor and the correspondingly reduced electrotonic length constant for the dendritic attenuation (Rall, 1977). Importantly, the degree of astrocytic neuronal enwrapping changes in an activity- and state-dependent manner during physiological challenges such as lactation and dehydration (Hatton, 1999; Langle et al., 2002; Theodosis et al., 2008; Panatier, 2009). Thus, we considered this a physiologically relevant biophysical parameter that can also influence somato-dendritic electrical coupling.

Overall, our results predict electrical compartmentalization or decoupling between MNN soma and dendrites, in which the presence of a varicosity, along with thinner dendritic stem diameters and restricted peri-dendritic space due to insulating glial wrapping, may restrict backpropagating of action potentials in these neurons.

## 2. Materials and methods

### 2.1. General approach

**FIGURE 1:**
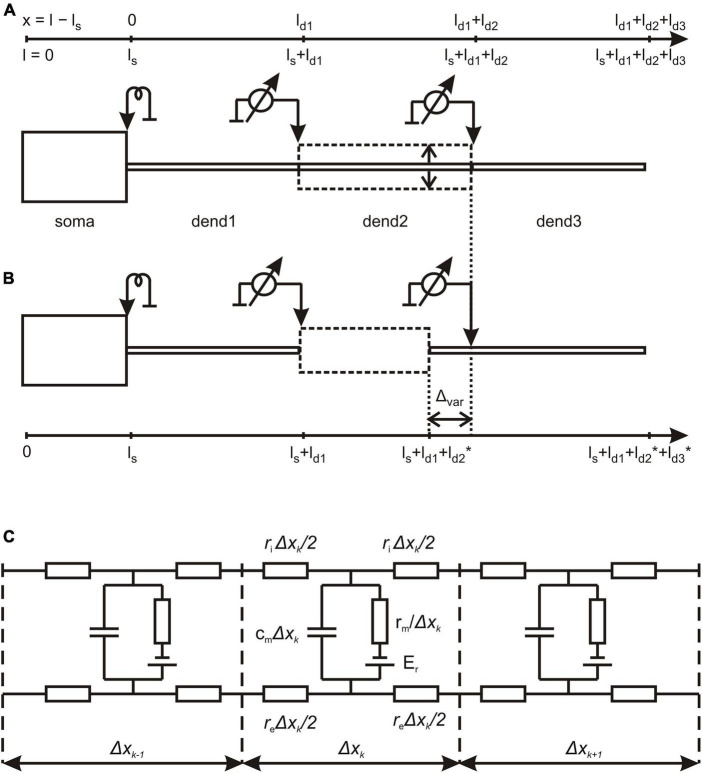
Structure of magnocellular neurosecretory neurons (MNN) models. **(A)** The reference configuration with dendritic compartments dend1, dend2, and dend3 of equal lengths and stem diameter (solid lines); dashed line indicates variation of dend2 diameter representing the varicosity. **(B)** The configuration, in which the varicose compartment dend2 has a varying length and the distal compartment dend3 has a co-varying length so that the total length of the three-compartment dendrite remains unchanged (see also Table 1). **(C)** The lamped equivalent electric circuit of dendritic cable with parameters per unit path length. Detailed explanations are given in the text.

Our approach to the problem of electrical compartmentalization (decoupling) in MNNs was based on the theory of electro-geometrical coupling (Korogod and Tyc-Dumont, 2009) and its parametric sensitivity (Korogod and Kaspirzhny, 2008, 2011) in biophysically complex dendrites. For that, we represented MNNs by linear mathematical models comprising of cylinder-shaped passive soma and dendrites (Figure 1).

The linear model is appropriate because (1) the linearization is a conventional first step in the analysis of non-linear systems, including neurons (Jack et al., 1975; Segev and London, 2000), and (2) electrical coupling between constituting parts of a neuron is performed *via* lateral currents flowing in *passive* conductive intra- and extra-cellular media (Jack et al., 1975; Korogod and Tyc-Dumont, 2009). The strength of electrical coupling between the soma and dendrite was quantified by the attenuation factor (AF) that is the ratio of the output voltage at a given dendritic site to the input (source) voltage at the soma: AF = V_*output*_/V_*input*_. Consequently, the output voltage is defined by the product of the input voltage and attenuation factor V_*output*_ = AF⋅V_*input*_. The smaller AF the smaller V_*output*_ and the greater attenuation. An alternative characteristic is the attenuation ratio that is the inverse value to the attenuation factor AR = 1/AF = V_*input*_/V_*output*_. The greater AR the smaller the output voltage and greater attenuation V_*output*_ = V_*input*_/AR.

In the linear model framework, we first characterized the general biophysical mechanisms that underlie the decoupling effects of MNN-specific micro geometry of the dendrites and peri-dendritic space and then estimated the domains of parameters, which promoted the decoupling. The voltage transfer from the somatic source to dendritic sites was represented by the longitudinal path profiles of the membrane potential. The transfers of steady voltages were defined as stationary (steady state) solutions to the MNN model equations. In general, these were piecewise uniform linear cable equations, for which the definitional domain comprised of 1D space and time intervals, *x*_*k*_ ∈ [0,*l*_*k*_] and *t* ∈ [0,τ], respectively, where *l*_*k*_ is the length of *k*-th section of somatic or dendritic cylinder and τ is the observation time. In each section of a uniform diameter *D*_*k*_, the membrane potential *E(x_k_, t)* was described by the equation (index *k* is omitted).


(1) $$\left(1/\left(r_{i}+r_{e}\right)\right)\cdot \partial^{2}E\,\left(x,t\right)/\partial x^{2}=c_{m}\cdot \partial E\,\left(x,t\right)/\partial t+g_{m}\cdot \left(E\,\left(x,t\right)-E_{r}\right)$$

where *E*_*r*_ is the resting membrane potential; *c*_*m*_ and *g*_*m*_ are, respectively, the membrane capacitance and conductance per unit cable length; *r*_*i*_ and *r*_*e*_ are, respectively, resistances of intracellular and extracellular media per unit cable length. These equations are supplemented by the initial conditions and boundary or coupling conditions. The initial condition was uniform rest potential *E(x*,0*) = E*_*r*_. At the junctions of consecutive *k*-th and (*k*+1)-th uniform sections, the coupling conditions were the membrane potential continuity *E*(*l*_*k*_, *t*) = *E*(*x*_*k*+1_ = 0, *t*) and the axial (core) current conservation *I*_*core*_(*l*_*k*_, *t*) = *I*_*core*_(*x*_*k*+1_ = 0, *t*). The latter equality is equivalent to setting the “leaky end” boundary condition at the end of *k*-th section such that its axial leak conductance equaled to the input conductance of (*k*+1)-th section: G_*Lk*_ = G_*inp,k*+1_. The most distal dendrite terminated with the “sealed end” boundary condition assuming zero axial current and therefore the voltage gradient: ∂*E*(*l*,*t*)/∂*x* = 0. The solutions *E*(*x*_*k*_, *t*) to the model equations were obtained either numerically in NEURON simulation environment (Carnevale and Hines, 2006) or derived analytically in terms of deviation from the rest potential: *V*(*x*_*k*_, *t*) = *E*(*x*_*k*_, *t*) *- E*_*r*_. The attenuation factors were computed as the ratio of steady voltages.


(2) AF(x)=V(x)/V(0)=(E(x)-E)r/(E(0)-E)r

In this approximation, two values of the attenuation factor *AF*_100_ measured at a path distance of 100 μm from soma were chosen as possible landmarks of the decoupling. The values of *AF*_100_ ≤ 0.1 and *AF*_100_ ≤ 0.01 corresponded to one- and two-order attenuated voltages, which were considered, respectively, insufficient for triggering dendritic spikes and undistinguishable from noise. 100 μm path distance from soma was chosen as characteristic recording location that is approximately 1/3 to 1/5 of typical 300–500 μm path lengths of the prototype MNN dendrites (Stern and Armstrong, 1998). This approach was implemented in multicompartment linear cable models to explore the decoupling roles of MNN-specific micro-geometrical features of the dendrites (stem diameters, varicosities) and peri-dendritic space (glia sheath).

### 2.2. Models

Magnocellular neurosecretory neurons models comprised of cylindrical soma and three-compartment dendrite (Figure 1A; Table 1).

**TABLE 1:** Electrical and geometrical parameters of the modeled magnocellular neurosecretory neuron (MNN) in Figure 1.

	Soma	dend1	dend2 (varicosity)	dend3
**Electrical parameters**				
Membrane capacitance, C_m_ (μF/cm^2^)	1	1	1	1
Membrane resistivity, R_m_ (Ohm⋅cm^2^)	1,000	1,000	1,000	1,000
Intracellular media resistivity, R_i_ (Ohm⋅cm)	200	200	200	200
Extracellular media resistivity, R_e_ (Ohm⋅cm)	100	100	100	100
**Geometry**				
Length, l (μm)	20	100	Varied 100 down to 10	Co-varied 100 up to 190
Diameter, d (μm)	20	2 or varied 2 to 0.01	2 or varied 2 to 10	2 or varied 2 to 0.01
Peri-dendritic layer thickness, Δ (μm)	Varied 100 to 0.001	Varied 100 to 0.001	Varied 100 to 0.001	Varied 100 to 0.001

#### 2.2.1. Geometry

The length *l*_*s*_ = 20 μm and diameter *d*_*s*_ = 20 μm of the soma were fixed. In the basic (reference) configuration (Figure 1A, solid lines), the dendritic compartments (dend1, dend2, and dend3) had equal lengths *l*_*d1*_ = *l*_*d2*_ = *l*_*d3*_ = 100 μm and diameters *d*_1_ = *d*_2_ = *d*_3_ = 2 μm that made a uniform 300 μm long dendrite. In other configurations, the middle compartment dend2 represented the varicosity of a greater diameter *d*_2_ > *d*_1_ = *d*_3_ than those of equally thin proximal and distal compartments dend1 and dend3 representing the dendrite stem. In configurations with the varicosity length reduced by a decrement (*l*_d2_* = *l*_*d2*_–Δ_*var*_), the distal compartment length was incremented by the same value (*l*_d3_* = *l*_*d3*_+Δ_*var*_), whereas the proximal compartment remained unchanged and so did the total dendrite length (Figure 1B). The above configurations were extended by adding a cylindrical layer of thickness (width) Δ representing peri-dendritic space between dendrite and glia sheath filled with conductive fluid.

#### 2.2.2. Electrical parameters

The model was characterized by a reference set of spatially uniform passive (linear) electrical parameters per unit membrane area, the capacitance *C*_*m*_ = 1 μF/cm^2^ and conductivity *G*_*m*_ = 1 mS/cm^2^ (or resistivity *R*_*m*_ = 1/*G*_*m*_) associated with the resting membrane potential *E*_*r*_ = -70 mV and the resistivity per unit volume of intracellular and extracellular conductive media, *R*_*i*_ = 200 Ohm⋅cm and *R*_*e*_ = 100 Ohm⋅cm, respectively. Corresponding parameters per unit length of uniform cable compartment were *c*_*m*_ = *C*_*m*_⋅π⋅*d*, *g*_*m*_ = *G*_*m*_⋅π⋅*d* or *r*_*m*_ = *R*_*m*_/(π⋅*d*), *r*_*i*_ = *R*_*i*_/(π⋅*d*^2^/4), and *r*_*e*_ = *R*_*e*_/(π⋅(Δ⋅*d*+Δ^2^)), where *d* is the compartment diameter. Noteworthy, *r*_*e*_ is negligibly small so that *r*_*e*_ < < *r*_*i*_ for *R*_*e*_*≈R*_*i*_ in case of excessively big Δ (large peri-dendritic space). Figure 1C shows the lamped equivalent electric circuit on example of three consecutive compartments of length Δ*x*_*k*_ with parameters per unit path length.

### 2.3. Protocols of simulation

An input steady voltage *V*_0_ is applied at the origin *x*_1_ = 0 of the proximal dendritic compartment dend1 and the output steady voltages *V*(*x*) are computed at different path distances *x* along the whole dendrite (*x* = *x*_*k*_ ∈ [0,*l*_*dk*_], *k* = 1, 2, and 3 along, respectively, dend1, dend2, and dend3).

### 2.4. Analytical steady-state solutions to the model equations

#### 2.4.1. Steady voltage transfer along non-uniform dendrite without glia wrapping

Equation (1) rewritten in terms of the transmembrane voltage as the deviation of the membrane potential from its resting value *V*(*x, t*) = *E*(*x, t*)–*E*_*r*_ is


(3) $$\left(1/\left(r_{i}+r_{e}\right)\right)\cdot \partial^{2}V\,\left(x,t\right)/\partial x^{2}=c_{m}\cdot \partial V\,\left(x,t\right)/\partial t+g_{m}\cdot V\,\left(x,t\right)$$

In the steady state and in the absence of glia sheath (large peri-dendritic space), *r*_*e*_ < < *r*_*i*_ it is reduced to


(4) $$\left(1/r_{i}\right)\cdot \left(\partial^{2}V\,\left(x\right)\right)/\left(\partial x^{2}\right)=V\,\left(x\right)/r_{m}$$

The membrane potentials along dend1 and dend2 (varicose) compartments are the solutions for leaky-end finite cables:


$$\begin{array}{l} V(x_{1})=V(x_{1}=0)\cdot (\cosh((l_{1}-x_{1})/\lambda_{1})+(G_{L1}/G_{\infty 1})\cdot \\ \sinh((l_{1}-x_{1})/\lambda_{1})))/ \end{array}$$

$$\left(\text{cosh}\,\left(l_{1}/\lambda_{1}\right)+\left(G_{L\,1}/G_{\infty \,1}\right)\cdot \text{sinh}\,\left(l_{1}/\lambda_{1}\right)\right),$$

$$\begin{array}{l} V(x_{2})=V(x_{2}=0)\cdot (\cosh((l_{2}-x_{2})/\lambda_{2})+(G_{L2}/G_{\infty 2})\cdot \\ \sinh((l_{2}-x_{2})))/ \end{array}$$

$$\left(\text{cosh}\,\left(l_{2}/\lambda_{2}\right)+\left(G_{L\,2}/G_{\infty \,2}\right)\cdot \text{sinh}\,\left(l_{2}/\lambda_{2}\right)\right),$$

whereas those along dend3 are the solutions for a sealed-end cable.


$$V(x_{3})=V(x_{3}=0)\cdot \cosh((l_{3}-x_{3})/\lambda_{3})/\cosh(l_{3}/\lambda_{3}).$$

These solutions are coupled at the inter-compartment junctions according to the voltage continuity conditions:


$$V(x_{1}=0)=V(x_{s}=l_{s})=V_{0}$$

$$V(x_{2}=0)=V(x_{1}=l_{1})\cdot 1/(\cosh\;(l_{1}/\lambda_{1})+(G_{L1}/G_{\infty 1})$$

$$\cdot \text{sinh}\left(l_{1}/\lambda_{1}\right)),$$

$$V(x_{3}=0)=V(x_{2}=l_{2})\cdot 1/(\cosh\;(l_{2}/\lambda_{2})+(G_{L2}/G_{\infty 2})$$

⋅sinh(l2/λ)2).

The leak conductance at the end of each preceding compartment equals to the input conductance of the following compartment, which is either “leaky-end” (dend1 and dend2) or “sealed-end” cable:


$$G_{i\,n\,p\,1}=G_{\infty \,1}\cdot \left(\text{tanh}\left(l_{1}/\lambda_{1}\right)+G_{L\,1}/G_{\infty \,1}\right)/(1+\left(G_{L\,1}/G_{\infty \,1}\right)$$

$$\cdot \text{tanh}\left(l_{1}/\lambda_{1}\right)),$$

$$G_{i\,n\,p\,2}=G_{\infty \,2}\cdot \left(\text{tanh}\left(l_{2}/\lambda_{2}\right)+G_{L\,2}/G_{\infty \,2}\right)/(1+\left(G_{L\,2}/G_{\infty \,2}\right)$$

$$\cdot \text{tanh}\left(l_{2}/\lambda_{2}\right)),$$

$$G_{i\,n\,p\,3}=G_{\infty \,3}\cdot \text{tanh}\,\left(l_{3}/\lambda_{3}\right),$$

where characteristic conductances are


$$G_{\infty \,s}=\left(\pi /2\right)\,\sqrt[{\frac{3}{2}}]{d_{s}}\,\sqrt[{\frac{1}{2}}]{G_{m\,s}/R_{i\,s}},$$

$$G_{\infty \,1}=\left(\pi /2\right)\,\sqrt[{\frac{3}{2}}]{d_{1}}\,\sqrt[{\frac{1}{2}}]{G_{m\,1}/R_{i\,1}},$$

$$G_{\infty \,2}=\left(\pi /2\right)\,\sqrt[{\frac{3}{2}}]{d_{2}}\,\sqrt[{\frac{1}{2}}]{G_{m\,2}/R_{i\,2}},$$

$$G_{\infty \,3}=\left(\pi /2\right)\,\sqrt[{\frac{3}{2}}]{d_{3}}\,\sqrt[{\frac{1}{2}}]{G_{m\,3}/R_{i\,3}},$$

and space constants are


$$\lambda_{s}=\sqrt{r_{m\,s}/r_{i\,s}}=\sqrt{\left(R_{m\,s}\cdot d_{s}\right)/\left(4\cdot R_{i\,s}\right)},$$

$$\lambda_{1}=\sqrt{r_{m\,1}/r_{i\,1}}=\sqrt{\left(R_{m\,1}\cdot d_{1}\right)/\left(4\cdot R_{i\,1}\right)},$$

$$\lambda_{2}=\sqrt{r_{m\,2}/r_{i\,2}}=\sqrt{\left(R_{m\,2}\cdot d_{2}\right)/\left(4\cdot R_{i\,2}\right)},$$

$$\lambda_{3}=\sqrt{r_{m\,3}/r_{i\,3}}=\sqrt{\left(R_{m\,3}\cdot d_{3}\right)/\left(4\cdot R_{i\,3}\right)}.$$

The attenuation factor at a characteristic 100-μm distance from soma is


$$AF_{100}=AF(x_{1}=l_{1})=V(x_{1}=l_{1})/V_{0}=1/(\cosh\;(l_{1}/\lambda_{1})+$$

$$\left(G_{L\,1}/G_{1}\right)\cdot \text{sinh}\left(l_{1}/\lambda_{1}\right)),$$

#### 2.4.2. Accounting for glia wrapping

In case of small peri-dendritic space (small Δ thickness/width of an interstitial layer of conductive fluid between the outer membrane of MNN and glia), the condition *r*_*e*_ < < *r*_*i*_ does not hold and the length constants for corresponding compartments are expressed [see e. g., Equation 2.18 in Rall (1977)] as:


$$\lambda_{\text{k}}=\sqrt{r_{\text{mk}}/\left(r_{\text{ik}}+r_{\text{ek}}\right)},$$

where *r*_mk_ = *R*_m_/(π⋅*d*_k_), $r_{\text{ik}}=R_{\text{i}}/\left(\pi \cdot d_{\text{k}}^{2}/4\right)$, and $r_{\text{ek}}=R_{\text{e}}/\left(\pi \cdot \left(\Delta_{k}\cdot d_{k}+\Delta_{\text{k}}^{2}\right)\right)$, k = s, 1, 2, 3 is the compartment index.

## 3. Results

The electrical compactness of a dendrite is estimated in terms of electrotonic length that is the ratio of its geometric path length *l* to the electrotonic length constant λ*: L = l*/λ. The greater λ the smaller the ratio *l*/λ, the smaller *L* and the more compact is the dendrite electrically. The latter also means smaller voltage attenuation with distance along the dendrite. Varicosed dendrites have sections of noticeably increased diameter that exemplify geometrical non-uniformity. In mathematical models, such non-uniform structures are represented by a sequence of uniform cylindrical sections with abrupt changes in diameter at their junctions and are called piecewise uniform. In a piecewise uniform dendrite, each uniform “piece” (section) is characterized by its own λ. The length constant λ generally includes micro-geometrical parameters of both the dendrite (diameter *d*) and the peri-dendritic space (thickness of conductive layer between the dendrite and glia sheath Δ; provided that Δ is essentially small). Therefore, we first explored the de-compacting effects of uniform and non-uniform (varicose) dendritic diameters and then those of peri-dendritic layer Δ.

### 3.1. Effects of stem diameter and presence of a varicosity on dendritic voltage attenuation

**FIGURE 2:**
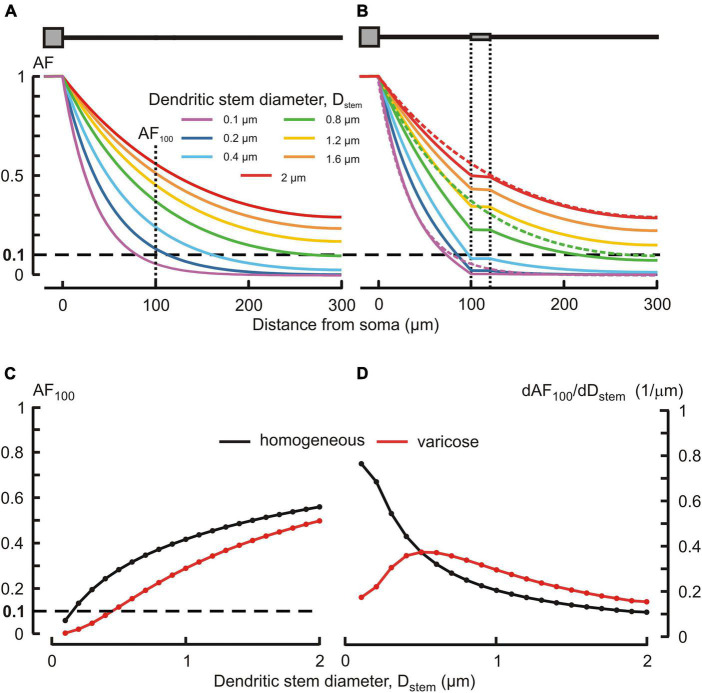
Effects of the dendritic stem diameter and presence of varicosity on the voltage attenuation along the dendrite. **(A,B)** Attenuation factor AF(x) (ordinate, dimensionless) as a function of the path distance from soma x (abscissa, μm) for different dendritic stem diameters *D*_stem_ (color-coded) in, respectively, the absence and presence of a varicosity of 20 μm length and μm diameter. Dotted lines in **(B)** represent plots of corresponding color in **(A)**. **(C)** Attenuation factor at 100 μm from soma *AF*_100_ (ordinate, dimensionless) as a function of the dendrite stem diameter *d*_1_ = *d*_3_ = *D*_stem_ (abscissa, μm). **(D)** Sensitivity function of the attenuation factor dAF_100_/dD_stem_ (ordinate, 1/μm) to changes in the *D*_stem_ (abscissa, μm) of homogeneous (black) and varicose (red) dendrite.

Typically, MNN dendrites are about 300–500 μm long, may have diameters of ∼2–3 μm at the origin from soma and are thinning to the limit of light microscope resolution (∼0.1 μm) at the distal ends. According open data on reconstructed dendritic morphology of mouse OT-ergic PV (Chen et al., 2022)^1^ and that of other hypothalamic neuron type (Scharbarg et al., 2016)^2^, the dendritic diameter at the origin can be as thin as ∼1 μm. Varicose extensions several times thicker than the stem are characteristic of MNN dendrites (Stern, 2001; Ludwig and Stern, 2015). In our simulated three-compartmental dendrite (Figure 1), the proximal and distal compartments (dend1 and dend3, respectively) corresponded to the stem and the middle one (dend2) corresponded to the varicosity. As a reference case, we considered somatofugal steady voltage attenuation along a uniform dendrite of total length *l*_*tot*_ = *l*_1_+ *l*_2_+ *l*_3_ = 300 μm with all compartments equally thick *d*_1_ = *d*_2_ = *d*_3_ = *D*_*stem*_ = 2 μm and with the uniform membrane and intracellular media resistivity of 1 kOhm⋅cm^2^ and 200 Ohm⋅cm, respectively (Figure 2A, red line). Reducing the diameter *D*_*stem*_ from 2 to 0.1 μm reduced the length constant λ from 158.11 to 35.36 μm and correspondingly increased the steepness of steady voltage attenuation (same figure, color-coded lines). Consequently, the attenuation with a factor of 0.1 (i.e., more voltage attenuation) was reached at progressively shorter path distances from the input (soma) (Figure 2A, color lines, and Figure 2C). For instance, at 100 μm from soma (end of the proximal compartment of interest) such attenuation occurred if *D*_*stem*_ was reduced to 0.15 μm, and in case of the thinnest tested *D*_*stem*_ = 0.1 μm the most proximal site of 0.1 attenuation was *x* = 81 μm (Figure 2A, line “0.1 μm” and Figure 2C).

Introducing a 20 μm long 6 μm thick varicosity in the middle compartment (top insert in Figure 2B) led to noticeably greater voltage attenuations than observed along uniform dendrites of corresponding stem diameters (cf. Figures 2A, B, plots of same color). In the proximal compartment, the attenuation factor *AF*_100_ monotonously decreased with decreasing *D*_*stem*_ in both uniform and varicose cases (Figure 2C, black and red plots, respectively), being always smaller (i.e., greater voltage attenuation) in the latter case. Introducing the varicosity in the thinnest tested dendrite (*D*_*stem*_ = 0.1 μm) shifted the most proximal site of 0.1 attenuation closer to soma, from *x* = 81 to *x* = 72.5 μm (Figure 2B, line “0.1 μm”). The biophysical reason for that is explained by the “leaky-end” boundary condition for the proximal compartment. Greater attenuation is due to smaller current through the proximal compartment membrane and correspondingly greater core current leak into the varicose compartment as compared to that into uniform continuation. Increased leak at the proximal compartment end is provided by a greater input conductance (smaller input resistance) of the varicosity because the cross-section area of the latter is greater than that of the dendritic stem. The *AF*_100_ sensitivity to monotonous decrease of *D*_*stem*_ was monotonously increasing in case of the uniform dendrite, but in the varicose case it was bell-shaped with the maximum near *D*_*stem*_ = 0.5 μm (Figure 2D, black and red plots, respectively).

### 3.2. Combined effects of varicosity diameter and length on dendritic voltage attenuation

**FIGURE 3:**
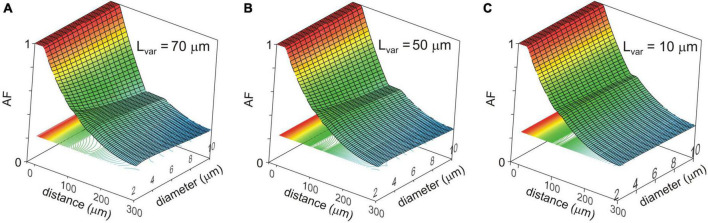
Surface and contour plots of the color-coded attenuation factor (AF) (applicate, dimensionless) in function of the path distance from soma x (abscissa, μm) and varicosity diameter D_var_ (ordinate, μm) for different varicose section lengths L_var_ = 70, 50, and 10 μm **(A–C)**. The AF range is subdivided into approximately 100 levels represented by the correspondingly colored contour plots in XY plane.

**FIGURE 4:**
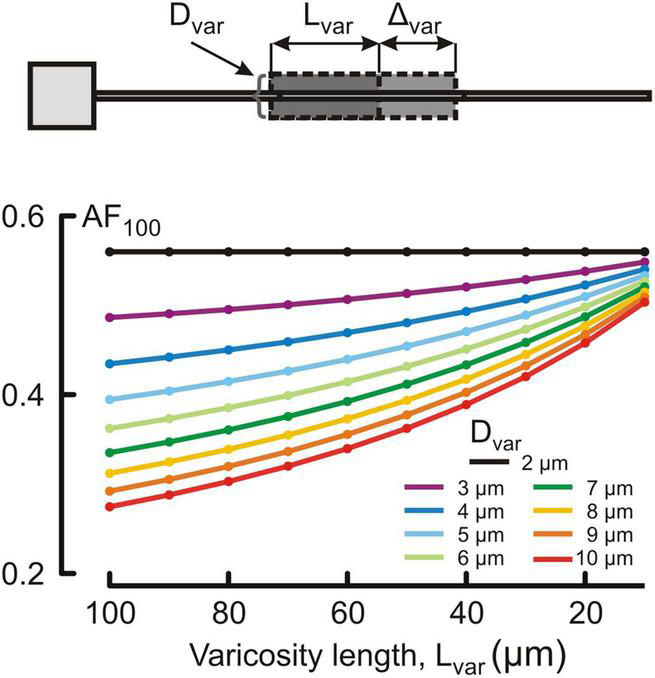
Dependence of the voltage attenuation factor at 100 μm from soma *AF*_100_ (ordinate, dimensionless) on the length *L*_var_ of the varicose section dend2 (abscissa, μm) for different varicosity diameters *D*_var_ (μm, color coded).

Changes in the attenuation factors *AF*(*x*) with the path distance *x* along the non-uniform dendrite (fixed reference stem diameter *D*_*stem*_ = 2 μm) were computed for various combinations of the length *L*_*var*_ and diameter *D*_*var*_ of the varicose compartment. *L*_*var*_ and *D*_*var*_ varied in the ranges of 10–100 μm and 2–10 μm, respectively. Typical relations are shown in Figures 3A–C for the varicosity lengths *L*_*var*_ = 70, 50, and 10 μm. The same relationships are represented in finer details by the attenuation factors computed at the end of the proximal compartment in 100 μm from soma *AF*(*x* = 100 μm) = *AF*_100_ in Figure 4. From Figures 3, 4, the following combined effects of varicosity diameter and length (dimensions) on the voltage attenuation in the proximal dendritic domain can be observed. On the one hand, for a given varicosity length, the thicker the varicosity the greater the voltage attenuation along the dendrite. On the other hand, for a given varicosity diameter, the shorter the varicosity the smaller the voltage attenuation. Biophysical explanations for these phenomena are provided below in the Discussion section.

### 3.3. Effects of peri-dendritic microgeometry

**FIGURE 5:**
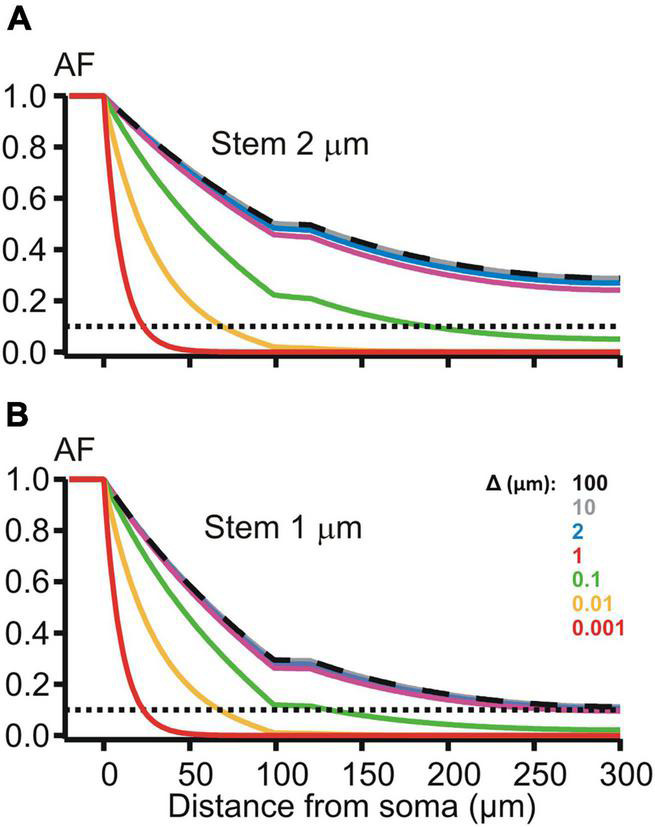
Attenuation factor AF(x) (ordinate, dimensionless) in function of the path distance coordinate x (abscissa, μm) for different width/thickness Δ (μm, color-coded) of the peri-dendritic layer of the interstitial conductive fluid between non-uniform dendrite and adjacent glia. **(A,B)** Plots for dendrites, which had the stem diameter of 2 and 1 μm, respectively, and contained a varicosity (length 20 μm, diameter 6 μm) starting at 100 μm from the soma.

The de-compacting effect of restricted peri-dendritic interstitial space between outer membrane of the dendrite and adjacent glial filaments (wrapping by glia) was explored on MNN models of the same geometry as that used in the previous experiments (Figure 2B), but with different expression for the length constant $\lambda_{\text{k}}=\sqrt{r_{\text{mk}}/\left(r_{\text{ik}}+r_{\text{ek}}\right)}$, k = s, 1, 2, 3 is the compartment index (see above). λ_*k*_ now included the extracellular (peri-dendritic) resistance per unit cable length $r_{\text{ek}}=R_{\text{e}}/\left(\pi \cdot \left(\Delta_{k}\cdot d_{k}+\Delta_{\text{k}}^{2}\right)\right)$, which depended on the thickness Δ_*k*_ of cylindrical layer of the peri-dendritic fluid (specific resistivity *R*_*e*_) surrounding each *k*-th compartment. Here we assumed the same layer thickness for all compartments Δ_*k*_ = Δ. Path distance profiles of the attenuation factor *AF*(*x*) along such glia-wrapped 2 μm thick dendrite for Δ = 100 and 10 μm (Figure 5, top panel, dashed black and solid gray lines) practically did not differ from each other and from that in case of non-wrapped dendrite surrounded by infinitely large peri-dendritic space (cf. Figure 2B, red line). Noticeable increase in the somatofugal voltage attenuation began when Δ reduced below 1 μm as illustrated in Figure 5 by increasing sag of green, yellow, and red lines (Δ = 0.1, 0.01, and 0.001 μm, respectively). At given Δ-*s*, the attenuation effects were stronger when the stem diameter was thinned from 2 to 1 μm (cf. top and bottom panels in Figure 5). The difference in the attenuation between thick and thin dendrites became small or almost indiscernible when the thickness Δ reduced to 0.01 μm and below (cf. yellow and red lines on the top and bottom panels of Figure 5). These observations indicate different sensitivity of the attenuation factor to changes of the peri-dendritic space thickness in different ranges.

**FIGURE 6:**
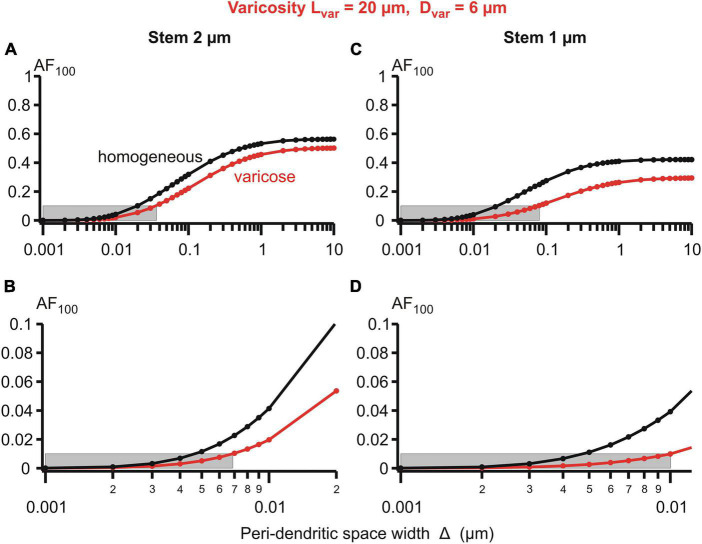
Attenuation factor AF_100_ (ordinate, dimensionless) in function width/thickness Δ (logarithmic abscissa, μm) of the peri-dendritic layer of the interstitial conductive fluid between heterogeneous dendrite and adjacent glial cells. Bottom plots are fragments of top plots. Dendrite of stem diameter 2 (left) or 1 μm (right) without (black plots) or with (red plots) varicosity of a fixed size (length 20 μm, diameter 6 μm) located in 100 μm from the dendrite origin. Grey boxes indicate 0.1 **(A,C)** and 0.01 **(B,D)** levels of *AF*_100_.

Details of the parametric sensitivity of the voltage attenuation to changes in the peri-dendritic space width are well-demonstrated by the semi-logarithmic plots of the attenuation factor *AF*_100_ vs. Δ for the given dimensions of the varicosity and dendritic stem diameters (Figure 6). The Δ-dependence of the *AF*_100_ is S-shaped: the attenuation is relatively low-sensitive to Δ changes in the upper (above ∼1 μm) and lower (below ∼0.01 μm) ranges, and is extremely sensitive in the intermediate range (approximately between 0.08 and 0.8 μm). As a consequence of such dependence, in the varicose dendrite *AF*_100_ drops below the first reference level of 0.1 if Δ is decreased below 0.04 μm at the stem diameter of 2 μm and below 0.08 μm at the stem diameter of 1 μm (Figure 6, upper panels). In the same dendrites, the respective values of Δ at which *AF*_100_ reaches the second reference level of 0.01 are 0.007 and 0.01 (same figure, lower panels).

### 3.4. Effects of the membrane conductivity (resistivity)

For each k-th compartment the electrotonic length constant λ_*k*_ depends on both geometrical (diameter *d*_*k*_, width of interstitial layer Δ_*k*_) and specific electrical (membrane resistivity per unit area *R*_*m*_, resistivity of intra- and extracellular media *R*_*i*_ and *R*_*e*_) parameters. Variation of these parameters, individually and in combinations, allows disclosing how the biophysical mechanism of electro-geometrical coupling (Korogod and Tyc-Dumont, 2009) contributes to functional compartmentalization of structurally heterogeneous dendrites.

**FIGURE 7:**
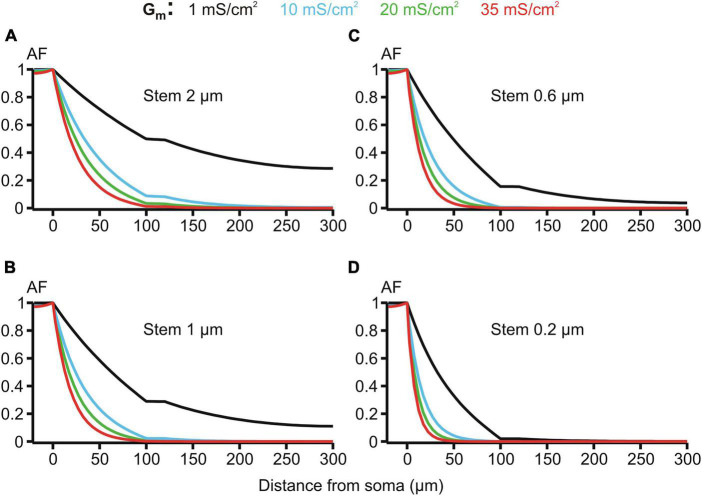
Attenuation factor AF(x) (ordinate, dimensionless) in function of the path distance from soma x (abscissa, μm) for different values of the membrane conductivity *G*_m_ = 1, 10, 20, and 35 mS/cm^2^ (color-coded) of the dendrites having different stem diameters *D*_stem_ = 2, 1, 0.6, and 0.2 μm **(A–D)** and the same varicosity (length 20 μm; diameter 6 μm).

The membrane resistivity *R*_*m*_ (or its inverse, conductivity *G*_*m*_ = 1/*R*_*m*_) is of special interest because, unlike *R*_*i*_ and *R*_*e*_, it may vary in a wide range depending on the activation of voltage- and ligand-gated ion conductances present in the MNN membrane. Such activity-dependent variation makes *R*_*m*_ a good candidate factor of dynamic modulation of microgeometry-induced de-compacting (decoupling) effects. One order increase in the uniform membrane conductivity *G*_*m*_ from its reference value of 1 mS/cm^2^ (*R*_*m*_ = 1 kOhm⋅cm^2^) to 10 mS/cm^2^ caused significant increase in the voltage attenuation along non-uniform dendrites of different stem diameters with the same fixed varicosity (20 × 6 μm) (cf. black and blue lines in Figure 7. With further increase in *G*_*m*_ to 20 and 35 mS/cm^2^ the voltage attenuated below the reference “decoupling” levels of 0.1 and 0.01 at progressively shorter path distances from soma (same figure, green and red lines, respectively) within the proximal dendritic compartment of interest. Data summarized in Table 2 shows that combinations of greater *G*_*m*_ and thinner stem diameters are favorable for dropping the attenuation factor *AF*_100_ at the end of the proximal dendritic compartment below the decoupling levels 0.1 and 0.01. Collectively, Figure 7, Table 2 demonstrate that increased *G*_*m*_ significantly augments the de-compacting effects of thinner stem diameters. This essentially expands the range of dendritic diameters for which the attenuation in the proximal dendrites reaches the levels critical for the decoupling.

**TABLE 2:** Attenuation factor AF_100_ at the end of the proximal compartment of the dendrites having different stem diameters and the membrane conductivities.

Stem diameter (μm)	Attenuation factor AF_100_ at a given membrane conductivity			
	**1 mS/cm^2^** **(reference case)**	**10 mS/cm^2^**	**20 mS/cm^2^**	**35 mS/cm^2^**
2.0	0.498	0.0872	0.0332	0.0161
1.0	0.39	0.0223	**0.0055**	**0.002**
0.6	0.16	**0.0056**	**0.0009**	**0.0003**
0.2	0.0197	**0.0001**	**0**	**0**
0.1	**0.0033**	**0**	**0**	**0**

Subcritical *AF*_100_ < 0.01 are in bold.

## 4. Discussion

Elucidating structure-dependence of electrical communication between axosomatic spike initiation zone and dendritic release sites of MNN is important for understanding the biophysical mechanisms contributing to somatodendritic release of neuropeptides from these neurons (Ludwig and Leng, 2006; Ludwig and Stern, 2015; Brown et al., 2020). It is generally assumed that somatodendritic release of OT and VP necessitates backpropagating APs. Still, dendritic release has been shown to occur in the absence of action potentials (Son et al., 2013; Pitra et al., 2019) and antidromic stimulation of axons in the pituitary failed to evoke dendritic release of VP (Ludwig et al., 2005).

To unveil specific roles of the dendritic varicosities, stem diameter, and peri-dendritic glial sheath played individually and collectively in the somato-dendritic electrical communication in MNNs, we employed electric circuits theory. In the framework of this theory, the coupling of circuit elements is described in terms of the transfer functions relating voltages and currents at certain elements considered the input and output (Korogod and Tyc-Dumont, 2009). In these terms, failure of somatic APs to backpropagate into the dendrites is interpreted as result of critically strong attenuation of the input signal generated at the soma when it is transferred to dendritic sites, the outputs.

Generation and propagation of APs in prototype MNNs are due to voltage-gated ion channels populating the cell membrane and thus endowing it with the non-linear electrical properties. Studies of complex non-linear systems conventionally start with their linearization (Jack et al., 1975; Segev and London, 2000; Cheng et al., 2010). Such approach is the golden standard as it provides a reasonable simplification, which bears and highlights the essential properties of the system underlying features of the non-linear processes. In neurons, including MNNs, electrical coupling between structural elements (axon, soma, dendrites) is performed by lateral currents, which flow in the intracellular and extracellular spaces (ICS and ECS, respectively) through conductive fluids having purely passive, linear ohmic properties. It should be noted that passive, linear models of neurons not only provide a standard initial approximation, but they are also directly relevant in particular neurophysiological events, such as subthreshold synaptic integration and dendritic release of neurotransmitter when the passive properties of the dendrites regulate the spread of the EPSP and thus the resulting Ca^2+^ transients (Roth and Häusser, 2005).

The ICS and ECS are shaped by the microgeometry of the neuron under study and surrounding neuronal and non-neuronal cells, including glia. Based on available data describing MNN microgeometry (Smith and Armstrong, 1990; Stern and Armstrong, 1998; Chen et al., 2022), we built biologically inspired cell models and explored geometry-related features of the somato-dendritic passive transfer of voltages and currents. To derive and solve model equations we used methods of the theory of electro-geometrical coupling and parametric sensitivity of the dendritic transfer functions, which were systematically described earlier (Korogod and Kaspirzhny, 2008, 2011; Korogod and Tyc-Dumont, 2009). With this approach, our model predicts critical features of MNNs dendrites, including heterogeneity of diameter accentuated by varicosities as well as restricted peri-dendritic space sculptured by glial environment to result in critically strong somato-dendritic attenuation of electrical signals. This strong attenuation of the somatic AP likely results in a depolarization at putative sites of dendritic release that is insufficient for triggering neuropeptide secretion. As described above, the quantitative criteria for the criticality were attenuation factors below 0.1 and 0.01. The two-order attenuated signal (factor 0.01) practically is drowned in electrophysiological noise. The one-order attenuation (factor 0.1) makes the output signal insufficient for triggering dendritic spikes because the depolarization shift does not sufficiently activate inward currents. For example, PVN neurons typically have resting potential in the range of –55 to –60 mV, the APs threshold of –35 to –40 mV and amplitude of 75 to 80 mV (Stern, 2001). Correspondingly, to reach the AP threshold the membrane potential must be shifted by about 20 mV from the rest. The one-order attenuation of the peak depolarization gives the shift of 7.5 to 8 mV that is 2.5-fold smaller than required 20 mV (even if one assumes dendritic spiking mechanism as strong as somatic).

To implement the above-mentioned approach, we first have chosen structurally uniform dendrites as a reference case and determined the attenuation factors for dendritic stem diameters ranging from 2.0 to 0.1 μm. Expectedly, thinner dendrites had shorter electrotonic length constant λ and stronger somatofugal electrical attenuation (Figure 2A). The well-known biophysical reason for this is that, for given uniform specific membrane and cytoplasm resistivities *R*_*m*_ and *R*_*i*_, at each dendritic site the dendritic membrane and core resistance per unit length are inversely proportional to the first and second power of the diameter *r*_*m*_ = *R*_*m*_/(π⋅*d*) and *r*_*i*_ = *R*_*i*_/(π⋅*d*^2^/4) and their ratio *r*_*m*_/*r*_*i*_ determining the space constant λ decreases with decreasing diameter. Another important and unique feature of dendrites in MNNs is their varicosed nature (Stern, 2001; Ludwig and Stern, 2015; Roy et al., 2021). Neuropeptide-containing dendritic varicosities can reach up to 15 μm in diameter (Ludwig and Leng, 2006). However, to what extent varicosities affect propagation of electrical signals in dendrites of MNNs is completely unknown. To assess the effect of varicosities and their microgeometry on electrical attenuation, a varicosity of fixed size was introduced in the reference uniform dendrites of different stem diameter (Figure 2B). In other computation experiments, single varicosities of different diameters and/or lengths were inserted in a particular reference dendrite having fixed stem diameter (Figures 3, 4). On the one hand, for a given varicosity length, the thicker the varicosity the greater the voltage attenuation along the dendrite. The biophysical reason for that is as follows. A greater leak into thicker varicose compartment changes the relation between components of the total current flowing in the proximal compartment so that the core component (through the leaky end) increases, and the transmembrane component decreases and thus produces smaller membrane depolarization. On the other hand, for a given varicosity diameter, the shorter the varicosity the smaller the voltage attenuation. The corresponding biophysical reason is as follows. A smaller leak into shorter varicosity followed by correspondingly longer thin higher-resistive distal compartment has opposite consequence for the relation between components of the proximal compartment total current: the core component (through the less leaky end) decreases, and the transmembrane component increases and thus produces greater membrane depolarization.

For the above-described biophysical reasons, the presence of varicose enlargements underlies significantly greater somatofugal voltage attenuation along the varicose dendrite compared to that along the homogeneous one given the same stem diameter (Figure 2B). Under certain combinations of sizes of the dendritic stem and varicosity, the attenuation can be so strong that dendritic parts become electrically decoupled from the soma even if they are at relatively short distances from the cell body (see voltage profiles below the dashed line in Figure 2). Earlier the effect of varicosities on the dendritic voltage attenuation in the opposite, somatopetal direction was explored on a linear model of retinal amacrine cell in the context of signal transfer from distal synapses to the soma (Ellias and Stevens, 1980). The input and output were, respectively, at the distal dendritic end and soma. Based on comparison of somatopetal voltage profiles along the varicose and homogeneous dendrites, the authors suggested that the function of the varicosities on amacrine cell dendrites might be to electrically isolate the local circuits of the mentioned dendritic input and somatic output.

Magnocellular neurosecretory neurons, including their dendrites, are typically enwrapped by glial processes (Hatton, 1999; Theodosis et al., 2008). Importantly, the degree of glial coverage can vary in an activity-dependent manner, and in response to relevant physiological conditions of the system, e. g., osmotic challenge and/or lactation (Hatton, 1999; Langle et al., 2002; Theodosis et al., 2008). While this phenomenon has been well-characterized and is known to be functionally relevant for adaptive responses (Panatier, 2009), again the impact of dynamic glial coverage on propagation of electrical signals remained largely unstudied. Earlier works noted that excitability of neurons is influenced by restricted neuroglial ECS, because the latter forms a physical barrier for the volume transmission of chemical (concentration) signals, such as diffusion of ions, neurotransmitters, hormones (Syková, 2004; Theodosis et al., 2008). Obviously, the same barrier also influences volume transmission of electrical signals — flow of charges and consequences for the local membrane potentials. Curiously, this aspect of cellular neurophysiology was overlooked until now. Our study emphasizes this aspect of neuro-glial relations. The dynamic change in the glial coverage of MNNs was simulated by variation of thickness of the peri-dendritic interstitial space filled with conductive cerebrospinal fluid (Figures 5, 6). The effects were compared for the cases of wrapped and non-wrapped dendrites with a varicosity of fixed size and two stem diameters, thicker and thinner. The overall effect was a significantly greater attenuation compared to non-wrapped cases if the interstitial space was narrowed to 0.1 μm and thinner, and it was more pronounced in case of thinner stem diameter. The biophysical reason for that was significantly increased longitudinal resistance of thinner peri-dendritic layer of extracellular interstitial space that provided significant shortening contribution to electrotonic space constants of wrapped compartments $\lambda_{\text{k}}=\sqrt{r_{\text{mk}}/\left(r_{\text{ik}}+r_{\text{ek}}\right)}$.

This study was intentionally focused at MNNs microgeometrical features potentially affecting the dendritic release of neuropeptides due to critical somato-dendritic attenuation of APs initiated at the trigger zone. Similar questions could be posed in relation to MNNs axons, which are usually thinner than dendrites and are also contacted by astrocytes. The axonal aspect remained out of scope of this study because the axon hillock and initial segment are conventionally considered constituent parts of the trigger zone that is rich of fast Na^+^ channels and thus secure forward propagation of APs along the axonal transmission line, unlike proximal dendrites lacking such electrical booster due to relatively lower density of voltage gated channels.

Finally, the effect of the membrane conductivity was characterized (Figure 7). The greater conductivity the greater attenuation because of greater loss of current (charge) through the leakier dendritic membrane.

While we focused on some key morphometric parameters of MNNs and their dynamic interaction with surrounding astrocytes, we acknowledge that factors others than this could also contribute to electrical somato-dendritic uncoupling in these neurons, including a low density of Na^+^ channels on proximal dendrites.

Based on the results described above, our model predicts that in hypothalamic MNNs, their relatively thin dendritic stem diameter, presence of abundant varicosities, and a tight peri-dendritic glial sheath, all together constitute micro-geometrical factors that will result in robust attenuation of electrical signals propagating from the axon hillock down the dendrites, resulting in the electrical decoupling and compartmentalization of proximal dendrites from the somatic spike initiation zone. The decoupling effects of these microstructural features are provided *via* structure-specific biophysical mechanisms. Varicosities increase the attenuation along proximal dendrites by providing an increased current leak at the end of the proximal dendritic section. The glial sheath promotes greater attenuation by increasing longitudinal resistance of the interstitial peri-dendritic space. These decoupling effects are strengthened in case of thinner diameters of the dendritic stems and/or increased conductivity of the membrane.

It is important to acknowledge also that different types of neurosecretory neurons have very distinct morphometries, and likely different mechanisms and factors regulating propagation of electrical signals and dendritic release. For example, GnRH neurons display a unique bipolar morphology comprising a classical proximal dendrite, and a very elongated spiny process, termed a “dendron,” which functions both as a dendrite and an axon (Campbell et al., 2005; Herbison, 2021). Importantly, while action potentials can propagate in these dendrites (Iremonger and Herbison, 2012), a recent study supports functional electrical compartmentalization in these neurons as well, with electrical activity in the soma-proximal dendrite being essential for the LH surge, while autonomous activity in the distal “dendron” drives pulsatile GnRH secretion (Wang et al., 2020). Thus, these results together suggest that electrical and functional compartmentalization in different neurosecretory neurons could be achieved by different means. Undoubtedly, future studies applying a similar modeling approach to different neurosecretory neuronal types will be needed to determine more accurately how specific dendritic morphometries, and their interactions with the local astrocyte network, affect propagation of electrical signals.

In summary, we believe these results have very important implications because they help explain counterintuitive phenomena of dendritic release in MNNs, namely that of action-potential independent release, and supports the notion that action potentials in these neurons fail to backpropagate to dendritic compartments. Thus, our results supporting somato-dendritic electrical uncoupling in MNNs suggest that a mechanism other than backpropagating action potentials (yet to be determined) contributes to dendritic release. Finally and importantly, given that the micro-geometrical properties of dendrites in MNNs change in conditions of high hormonal demand (e.g., osmotic challenge, lactation), it is likely the degree of electrical coupling/decoupling between somatic and dendritic compartments varies accordingly during these conditions. Future experimental approaches using simultaneous somato-dendritic recordings will be needed to confirm the predictions of our model.

## Data availability statement

The original contributions presented in this study are included in the article/supplementary material, further inquiries can be directed to the corresponding author.

## Author contributions

SK developed mathematical and computer models, performed the computational experiments, and wrote the original manuscript. GC contributed to the design and development of this study and model. JS and GC coordinated the work and edited the manuscript. All authors conceived this work, revised, and contributed to the submitted version of the manuscript.

## Footnotes

1
https://neuromorpho.org/neuron_info.jsp?neuron_name=0126_S1C2

2
https://neuromorpho.org/neuron_info.jsp?neuron_name=VLPO-23
